# Validity and Reliability of the GymAware Linear Position Transducer for Squat Jump and Counter-Movement Jump Height

**DOI:** 10.3390/sports6040177

**Published:** 2018-12-19

**Authors:** Tanuj Wadhi, Jacob T. Rauch, Nauris Tamulevicius, Jody C. Andersen, Eduardo O. De Souza

**Affiliations:** Human Performance Lab, Department of Health Sciences and Human Performance, The University of Tampa, Tampa, FL 33606, USA; jacobrauch1@gmail.com (J.T.R.); ntamulevicius@ut.edu (N.T.); jcandersen@ut.edu (J.C.A.); edesouza@ut.edu (E.O.D.S.)

**Keywords:** vertical jump, fatigue monitoring, power assessments

## Abstract

The purpose of this study was to assess the concurrent validity and test-retest reliability of a linear position transducer (LPT) for the squat jump (SJ) and counter-movement jump (CMJ) height. Twenty-eight subjects (25.18 ± 7.1 years) performed three SJs followed by three CMJs using a force plate concurrently with the LPT to test validity. Subjects returned on a separate day, at least 48 h apart, to measure test-retest reliability. A *t*-test showed a significant difference between the two devices for both SJ (*p* < 0.001) and CMJ (*p* < 0.001) while Bland–Altman analysis for validity revealed that the LPT overestimated jump height for both SJ (mean difference (MD) = 8.01 ± 2.93 cm) and CMJ (MD = 8.68 ± 2.99 cm). With regards to reliability of the LPT, mean intraclass correlation (ICC) for both SJ (ICC = 0.84) and CMJ (ICC = 0.95) were high, and Bland–Altman analysis showed mean differences lower than minimal detectable change (MDC) between the days for both SJ (MD = 1.89 ± 4.16 cm vs. MDC = 2.72 cm) and CMJ (MD = 0.47 ± 3.23 cm vs. MDC = 2.11 cm). Additionally, there was a low coefficient of variation (CV) between days for both SJ (CV = 3.25%) and CMJ (CV = 0.74%). Therefore, while the LPT overestimates jump height, it is a reliable tool for tracking changes in jump height to measure performance improvement and monitor fatigue.

## 1. Introduction

The vertical jump is an important skill for a multitude of team sports, including volleyball and basketball, where jump height has been related to in-game performance [1,2,3,4,5,6]. Moreover, coaches can use performance on the squat jump (SJ) and counter-movement jump (CMJ) to assess neuromuscular status and recovery [7,8].

Linear position transducers (LPTs) are portable and practical tools that measure the displacement and velocity of an object using optical encoding technology [9]. LPTs have become common in strength and conditioning (S&C) facilities, due to the widespread adoption of velocity-based training [10,11]. In addition to prescribing training velocities, some LPTs also measure the distance traveled by the bar, and this function of the LPT can be used to assess vertical jump height prior to a training session to measure athlete readiness and recovery. Depending on the athlete’s score in relation to their norm, the S&C coach(es) can use this objective feedback to increase or decrease the prescribed load for a given session (i.e., autoregulation) [12,13]. However, this application of LPTs is only valuable insofar as the measurements are valid and reliable.

GymAware is a distinct type of LPT, as it considers the angle of the lift using a sensor near the bottom of the tether where it leaves the spool (GymAware; Kinetic Performance Technologies, Canberra, Australia). This allows the vertical-only displacement to be accurately measured by correcting for any horizontal movement using basic trigonometry [14], as opposed to previous generation LPTs that did not account for horizontal movement, and therefore overestimated the movement distance. Previous studies have looked at the force and power output of the GymAware and other LPTs during SJ and CMJ [15,16,17,18,19], however, only one study [20] has examined the validity and reliability of the GymAware LPT for measuring CMJ height. While the authors found that GymAware is reliable for measuring CMJ height (intraclass coefficient: 0.70), it may overestimate CMJ height performance (mean difference: 7.0 ± 2.8 cm) [20]. In addition, it should be noted that O’Donnell et al. used a homogeneous sample (i.e., female volleyball athletes), which may impose threats to the external validity and reliability.

Moreover, while the CMJ is common in sport, the SJ is also a valuable assessment for examining the rate of force development without the stretch shortening cycle [21]. To the best of our knowledge, there are no data available regarding the validity and reliability of the GymAware LPT for assessing SJ performance in relation to jump height, and it therefore needs to be determined against a “gold standard” (i.e., force plate) method to estimate its concurrent validity, and test-retest reliability. Therefore, the purpose of the present study was two-fold: (1) to assess the concurrent validity of the GymAware LPT as compared to the criterion assessment, the force plate, which is the current “gold standard” for vertical jump assessment [22,23], and (2) to determine the inter-day reliability of the GymAware LPT. Additionally, the subjects in this study comprised a heterogeneous sample as opposed to the homogeneous sample in the study by O’Donnell et al., as it is important to reassess the validity of GymAware in a more heterogeneous population with different jumping abilities and patterns [24] to overcome potential threats to the validity of SJ and CMJ performance.

## 2. Materials and Methods

### 2.1. Subjects

A total of 28 subjects (25.18 ± 7.10 years) volunteered to take part in the current study. A population diverse in terms of exercise experience (<12 months: *n* = 6; 6–12 months: *n* = 8; >12 months: *n* = 14), and age (range: 19–47 years) was recruited through the university’s “Health Science and Human Performance” department via word-of-mouth. To be eligible for the study, the subjects had to be free from lower-limb injury, joint pain, hypertension, or any other contraindications to vertical jumping. All 28 subjects were included into the study, but only eighteen (age: 22.11 ± 2.22 years) returned for the second day of testing, and only the 18 that returned were included for reliability analysis. Subjects were informed about the study design including potential risks and benefits, and were required to sign an informed consent prior to inclusion. This study was given ethical clearance by the university’s institutional review board committee.

### 2.2. Validity

A laboratory force plate served as the criterion device. Force data was recorded with AccuPower (Advanced Mechanical Technology Incorporated, Watertown, MA, USA), which uses the Hall effect to measure forces in all six axes, at a frequency of 1200 Hz for 6 s. Concurrently, the GymAware LPT (GymAware, Kinetic Performance Technology, Canberra, Australia) was attached onto the subjects via a waist belt (Keiser Corporation, Fresno, CA, USA) for every jump. The GymAware LPT uses an adaptive sampling rate and filters the output to remove noise [25,26].

### 2.3. Reliability

Subjects were asked to report back to the lab on a separate day at least 2 days apart in the same or similar clothing to improve internal consistency, and same or similar time of day to account for diurnal variation. The subjects repeated three SJs and three CMJs with the GymAware LPT attached via a waist belt to assess the test-retest reliability of the GymAware LPT.

### 2.4. Protocol

**Figure 1:**
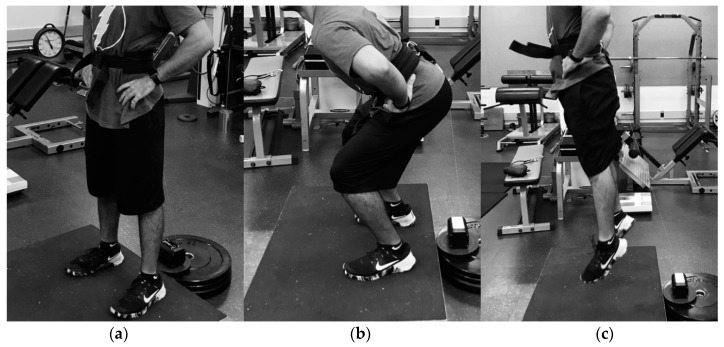
(**a**) Participant ready and standing in ready position, (**b**) participant in self-selected squat stance, and (**c**) participant in flight phase of the jump.

The protocol for the jump assessments was identical for both sessions. On arrival to the lab, the subjects performed a standardized warm-up on a Tuff Tread treadmill (White Phoenix LLC, Conroe, TX, USA) at 4.8 km/h for 3 min. Following the warm-up, subjects were weighed on the force plate, and hooked up to the GymAware LPT. The subjects then performed three SJs followed by three CMJs on the force plate with the GymAware LPT concurrently attached to the athlete via a waist belt. The waist belt was positioned to sit just above the iliac crest of the subjects to ensure a similar fit each time, and was tight enough so as to not move during the jumps. Subjects started in an upright position for both jumps, with hands placed on their hips throughout the jumping motion. All subjects were given the same verbal commands; for the SJs, two verbal commands were given: “set” to allow the subject to get into the self-selected half squat position and were given 3 s to stabilize; and “jump”, at which point they were instructed to jump as high as possible from the squat position without dipping. For the CMJs, a single “jump” command was given, where the subjects would dip into a self-selected half squat, and immediately jump as high as possible. The subjects were instructed to keep their legs straight during the flight phase of both the jumps [20]. A rest interval of ~30 s was given between each jump trial (Figure 1).

The GymAware LPT was tested for accuracy of velocity and displacement output prior to the first testing session and after the last testing session using the isokinetic function on a dynamometer (Primus RS, BTE Technologies, Hanover, MD, USA) at a speed of 30 degrees/s for a 120-degree rotation corresponding to 0.48 m in height at 0.12 m/s. The calibration testing on both occasions showed that the GymAware LPT was 100% accurate in both the tests (velocity and displacement), measuring a velocity of 0.12 m/s and displacement of 0.48 m.

The GymAware LPT was connected to an iPad (Apple Inc., Cupertino, CA, USA) via a wireless connection (Bluetooth 4.0, Bluetooth Special Interest Group, Kirkland, WA, USA) with the manufacturer’s app installed. The GymAware LPT was placed next to the force plate, and behind the subject, attached to a magnetic weight plate at the same height as the base of the force plate.

The LPT was recalibrated prior to each subject performing the jumps by “zeroing” the LPT while the tether was disconnected from the waist belt and fully retracted into the spool. Jump height from the LPT was determined based on change in displacement from the starting position (standing erect) to peak positive displacement (maximum jump height), and was recorded from manufactures software (GymAware v2.4.1, GymAware, Kinetic Performance Technology, Canberra, Australia). The manufacturer’s software (AccuPower 2.0, Advanced Mechanical Technology Incorporated, Watertown, MA, USA) uses the vertical component of the ground reaction force and applies the impulse-momentum theorem to estimate the concentric impulse and the take-off velocity used to calculate the jump height (h = v^2^/2g, where h is the jump height, v is the take-off velocity, and g is the acceleration due to gravity).

### 2.5. Statistical Analysis

The concurrent validity of GymAware LPT was assessed using a paired two-tailed *t*-test to check for a difference between the results from GymAware and the force plate following a Shapiro-Wilk test for normality. Additionally, 95% confidence intervals (CI) for the mean are also reported. Bland–Altman plots were used to represent the bias between force plate and GymAware LPT. In addition, the limits of agreement which shows the mean difference (MD) between the two methods is presented. The reliability of GymAware LPT was assessed via intraclass correlation coefficient (ICC) analysis using single measure, two-way mixed, absolute-agreement parameters [27]. The highest jump from each subject on day 1 and day 2, retrieved from the GymAware LPT, was used. Since ICC shows a measure of the variability between subjects’ results compared to the total variability, high values of the ICC coefficient indicate that the test might be reliable [28]. Bland–Altman plots and limits of agreement analysis were used to determine the variance in the values between the two days. Minimal detectable change (MDC), a statistical estimate of the smallest amount for the measurement to be considered a real change [29], was calculated as 1.96 * standard error of mean * √2. Coefficient of variation (CV) was calculated (standard deviation expressed as a percentage of the mean) between the means of the entire sample from day 1 and day 2 to represent typical error [30]. Significance level was set at *p* < 0.05. Statistical analyses were run on Prism (Prism 7 v7.02, GraphPad Software, La Jolla, CA, USA), and Excel (Microsoft Excel^®^ for Mac v15.40, Microsoft Corporation, Redmond, WA, USA).

## 3. Results

### 3.1. Validity

**Figure 2:**
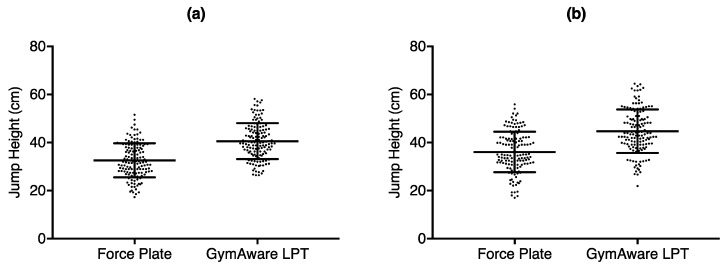
Mean difference of vertical jump height obtained from the force plate compared to the GymAware LPT for (**a**) squat jump height and (**b**) counter-movement jump height. Each point corresponds to each of the 3 jumps from each subject from both days.

**Figure 3:**
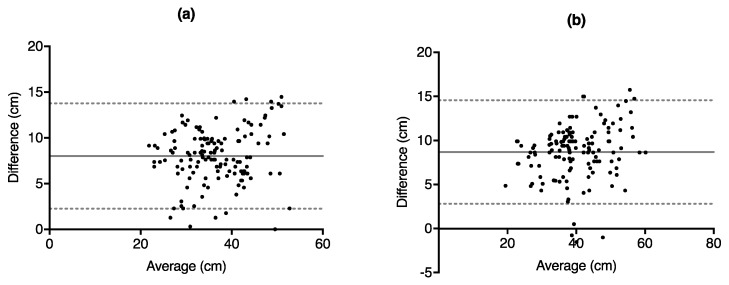
Bland–Altman difference vs. average plots depicting vertical jump height obtained from GymAware LPT—force plate vs. average of GymAware LPT and force plate for (**a**) squat jump height and (**b**) counter-movement jump height. Each point corresponds to each of the 3 jumps from each subject from both days.

Results from the paired *t*-test showed a statistically significant difference between the force plate and GymAware for both SJ (95% CI: 7.52 cm to 8.50 cm; *p* < 0.001) and CMJ (95% CI: 8.18 cm to 9.18 cm; *p* < 0.001), while Bland–Altman tests revealed that the GymAware LPT overestimated the jump heights for both SJ (mean difference (MD): 8.01 ± 2.93 cm) and CMJ (MD: 8.68 ± 3.00 cm). Table 1 highlights the results from the *t*-tests and Bland–Altman analysis, while Figure 2 and Figure 3 depict the Bland–Altman plots.

### 3.2. Reliability

**Figure 4:**
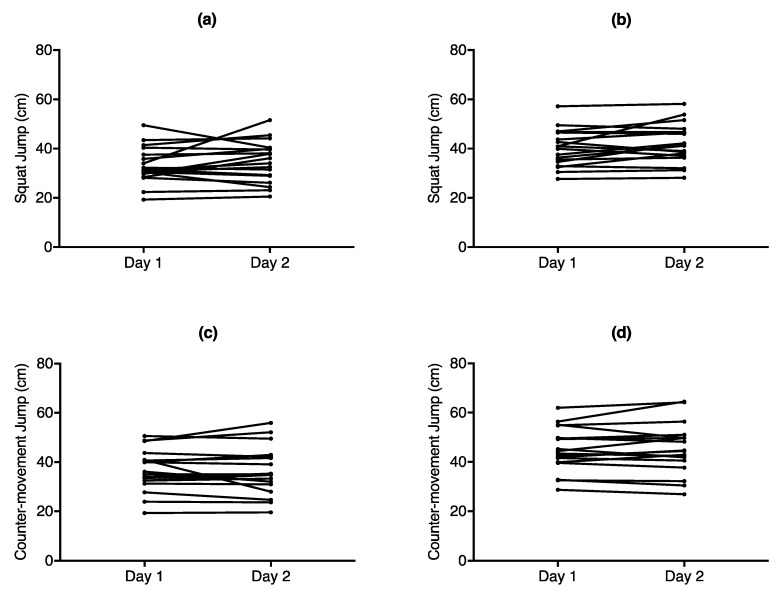
Line graphs to depict the difference of vertical jump height between days for (**a**) squat jump height derived from the force plate (**b**) squat jump height derived from the GymAware LPT (**c**) counter-movement jump height derived from the force plate (**d**) counter-movement jump height derived from the GymAware LPT. Each point corresponds to the highest of jump of returning subjects.

**Figure 5:**
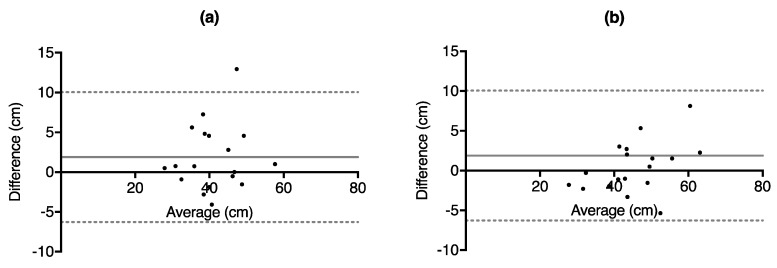
Bland–Altman difference vs. average plots depicting vertical jump height from day 2–day 1 vs. average of two days obtained from the GymAware LPT for (**a**) squat jump height (**b**) counter-movement jump height. Each point corresponds to the highest of jump of returning subjects.

ICC analysis revealed high correlation between day 1 and day 2 for SJ (ICC = 0.84) and extremely high correlation for CMJ (ICC = 0.95) [27], while the Bland–Altman analysis revealed that the SJ had a MD of 1.89 cm, while the CMJ had a MD of 0.47 cm between the days. Table 2 shows the results from ICC, Bland–Altman analysis along with MDC and CV between days, while Figure 4 and Figure 5 depict the Bland–Altman plots.

## 4. Discussion

The purpose of this study was twofold: (1) to assess the concurrent validity of the GymAware LPT as compared to the “gold standard” force plate, and (2) to determine the inter-day reliability of the GymAware LPT.

Regarding validity, *t*-test and Bland–Altman analysis revealed there was a systematic overestimation of jump height (MD = SJ: 8.01 cm; CMJ: 8.68 cm) by the GymAware LPT. Our results are in agreement with O’Donnell et al., who demonstrated an average overestimation of 7 ± 2.4 cm on the counter-movement jump height in female athletes. Therefore, absolute vertical jump heights extrapolated from the GymAware need to be used with precaution. While it is difficult to pinpoint the exact reasoning for the inflated jump heights, it could be due to the overestimation of other jump performance metrics (i.e., power and velocity) demonstrated by LPTs in previous investigations [16,17,18,19].

However, we found that the GymAware is very consistent, showing good test-retest reliability for the SJ (ICC = 0.84) and excellent test-retest reliability for the CMJ (ICC = 0.95), as described by Koo & Li (2016) [27], as well as low CV between days (SJ: CV = 3.24%; CMJ: CV = 0.74%), especially when the numbers are compared to the CV between days from the force plate (SJ: CV = 3.06%; CMJ: CV = 0.33%). Regarding differences between CV for CMJ and SJ, the CMJ is a more natural movement, and the participants probably were more accustomed to doing CMJs during their life while they were probably never familiarized with SJ. O’Donnell et al. showed similar results for CMJ height, albeit with slightly lower reliability (ICC = 0.70) than ours, strengthening the argument for the usage of GymAware as a monitoring tool.

Certainly, our study has limitations; even though our subjects were oriented to avoid strenuous exercise 24 h prior to the assessments, we were unable to standardize exercise protocols outside the study, and this could potentially have affected intra-subject performance.

In conclusion, we confirmed previous data that GymAware overestimates vertical jump height not just in athletes (e.g., female volleyball athletes), but also in a more heterogeneous population. It has however been shown to be valid for accurately measuring peak velocity during lifts [31] and can therefore be used to autoregulate load during strength-training based on athlete’s recovery level [32]. Nevertheless, the GymAware demonstrated a good test-retest reliability for jump height, and can therefore be used by S&C coaches and practitioners to monitor neuromuscular status and recovery [7,8], in real-time, prior to a scheduled lift, instead of taking time out to go to a laboratory setting to use a force plate. The ease of assessment alongside its reliability allows for frequent monitoring of athlete readiness and can be used to individualize training loads (i.e., autoregulation). Future research should aim at determining the mechanism behind the systematic overestimation of LPTs for assessing jump performance.

## Figures and Tables

**Table 1:** Results of tests comparing the vertical jump heights from the force plate and the GymAware LPT (mean ± standard deviation).

Jump	FP Height	LPT Height	MD	SEM of Diff	95% LOA	*t*-Test (*t*, df)	*t*-Test (*p*-Value)
SJ	32.59 ± 7.08 cm	40.6 ± 7.49 cm	8.01 ± 2.93 cm	0.25 cm	2.26–13.76 cm	32.42, 140	<0.001
CMJ	36.05 ± 8.44 cm	44.73 ± 9.01 cm	8.68 ± 3.00 cm	0.25 cm	2.81–14.56 cm	34.40, 140	<0.001

SJ = squat jump, CMJ = counter-movement jump, FP = force plate, LPT = GymAware LPT, MD = mean difference, SEM of Diff = standard error of the mean of mean difference, LOA = limits of agreement, df = degrees of freedom.

**Table 2:** Results of test-retest reliability for vertical jump height between testing days using the GymAware LPT (mean ± standard deviation).

Jump	D1 Height	D2 Height	MD	95% LOA	MDC	ICC	CV
SJ	40.14 ± 7.51 cm	42.03 ± 7.99 cm	1.89 ± 4.16 cm	−6.27 to 10.04 cm	2.72 cm	0.84	3.25%
CMJ	44.98 ± 8.86 cm	45.45 ± 10.25 cm	0.47 ± 3.23 cm	−5.86 to 6.80 cm	2.11 cm	0.95	0.74%

SJ = squat jump, CMJ = counter-movement jump, D1 = day 1, D2 = day 2, MD = mean difference, LOA = limits of agreement, MDC = minimal detectable change, ICC = intraclass correlation coefficient, CV = coefficient of variation.
